# Supplementary material for: Quantitative MRI of a Cerebral Cryptococcoma Mouse Model for In Vivo Distinction between Different Cryptococcal Molecular Types
Source: J Fungi (Basel). 2024 Aug 22;10(8):593. doi: 10.3390/jof10080593 (PMC11355240; doi:10.3390/jof10080593)
Supplement: Supplementary file 1 [file jof-10-00593-s001.zip › jof-3098812-supplementary.pdf]

*Supporting Information*

# Quantitative MRI of a Cerebral Cryptococcoma Mouse Model for In Vivo Distinction between Different Cryptococcal Molecular Types

**Luigi Musetta [1], Shannon Helsper [1], Lara Roosen [1], Dries Maes [1], Anca Croitor Sava [1], Liesbeth Vanherp [1,2], Willy Gsell [1], Greetje Vande Velde [1], Katrien Lagrou [3,4], Wieland Meyer [5] and Uwe Himmelreich [1,*]**

[1] Biomedical MRI, Department of Imaging and Pathology, KU Leuven, 3000 Leuven, Belgium
[2] µNEURO Research Centre of Excellence, University of Antwerp, 2000 Antwerp, Belgium
[3] Laboratory of Clinical Microbiology, Department of Microbiology, Immunology and Transplantation, KU Leuven, 3000 Leuven, Belgium
[4] Department of Laboratory Medicine, National Reference Center for Mycosis, UZ Leuven, 3000 Leuven, Belgium
[5] Westerdjjk Fungal Biodiversity Institute—KNAW, 3584 CT Utrecht, The Netherlands;
* Correspondence: uwe.himmelreich@kuleuven.be

**Supplementary Table S1. Characteristics of the *Cryptococcus neoformans and Cryptococcus gattii* isolates.** Cell and capsule size were determined by India ink stainings after growth on Sabouraud agar plates at 37°C for 4 days and scanning with a Zeiss Axio Scan Z.1 microscope. Cellular dimension were obtained by manual delineation using QuPath 0.4.3. Note: All isolates are considered standard/ reference strains for molecular typing according to Meyer et al. [1]. Additional characteristics, molecular information and other references to the used isolates can also be found in [1–3].

| Isolate. | MAT/ Serotype | Molecular type | Origin | Capsule thickness (µm) | Cell size (µm) |
|---|---|---|---|---|---|
| WM 148 | $\alpha$A | VN I | CSF, HIV-,Sydney (Australia), 1989 | 0.46 ± 0.12 | 5.87 ± 0.87 |
| WM 626 | $\alpha$A | VN II | CSF, Sydney (Australia), 1993 | 0.56 ± 0.11 | 5.29 ± 0.68 |
| WM 628 | $\alpha$A/ $a$D | VN III | CSF, HIV-,Sydney (Australia), 1988 | 0.48 ± 0.08 | 5.54 ± 1.04 |
| WM 629 | $\alpha$D | VN IV | Blood, HIV+, Melbourne (Australia), 1987 | 0.52 ± 0.12 | 6.64 ± 0.83 |
| WM 179 | $\alpha$B | VG I | CSF, HIV-,Sydney (Australia), 1993 | 1.60 ± 0.16 | 8.25 ± 0.67 |
| WM 04.71 | $\alpha$B | VG II | Cat, Sydney (Australia), 1991 | 1.37 ± 0.06 | 8.10 ± 0.43 |
| WM 183 | $\alpha$B | VG III | CSF, San Diego, California (USA), 1981 | 0.62 ± 0.15 | 5.35 ± 0.59 |
| WM 779 | $\alpha$C | VG IV | Cheetah, Johannesburg (South Africa), 1994 | 1.23 ± 0.29 | 6.63 ± 0.71 |

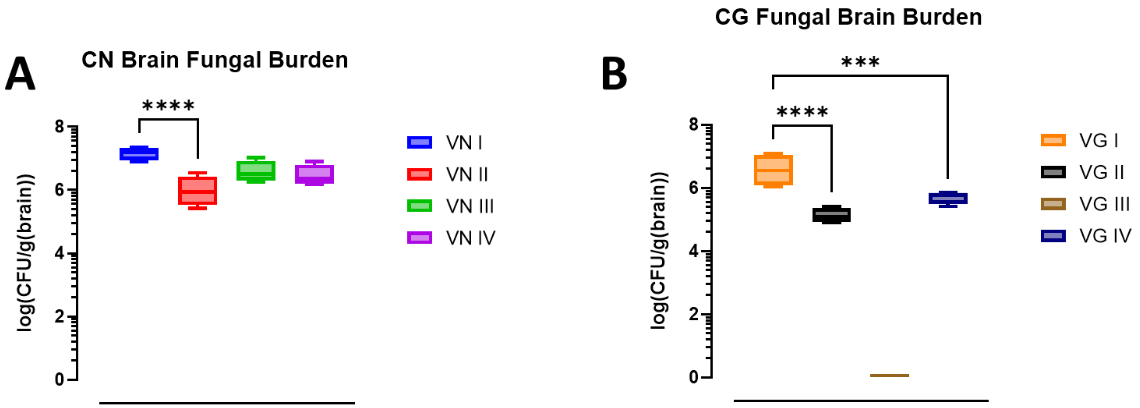


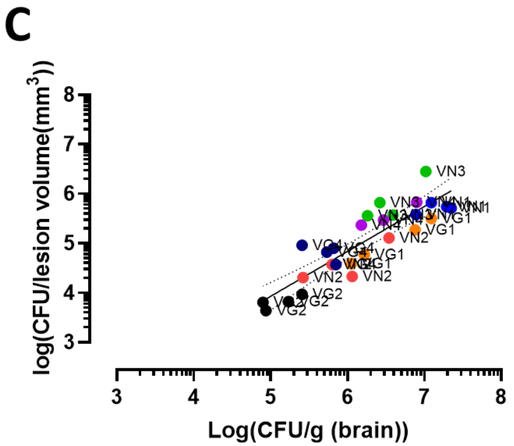


| | |
|---|---|
| r | 0.8830 |
| 95% confidence interval | 0.7569 to 0.9457 |
| R squared | 0.7796 |
| | |
| P value | |
| P (two-tailed) | <0.0001 |
| P value summary | **** |
| Significant? (alpha = 0.05) | Yes |
| | |
| Number of XY Pairs | 27 |

**Figure S1. Correlation of lesion density and fungal burden.** A. & B. CN and CG brain fungal burden differences across the different major molecular types. The graphs show median distribution and standard deviation. One way ANOVA with Fisher LSD test. (p-value, ***<0.001,****<0.0001) ; C. The graph is a linear Pearson correlation between lesion density and fungal burden in the whole brain.

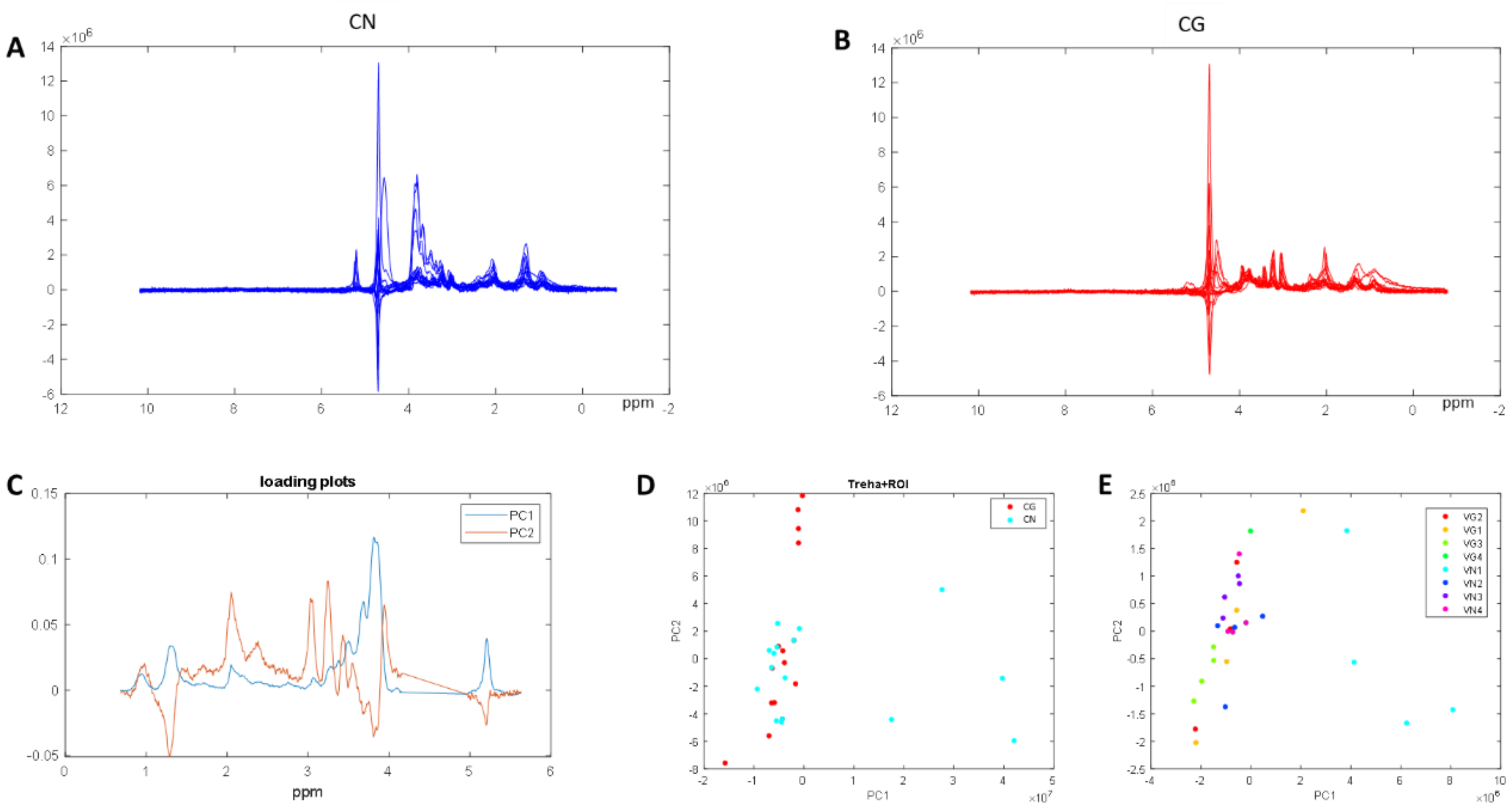


**Figure S2. Endpoint MR spectroscopy data for representative isolates of CN and CG, and principal components analysis of MRS data.** MR spectra at endpoint obtained from CN (A) and CG (B) representative isolates. Principal components analysis (PCA) loading plots for PC1 and PC2 are shown (C), together with their coefficients. The distribution of species related (D) and isolate related (E) in the PCA coefficient space of PC1 and PC2 is colour marked.

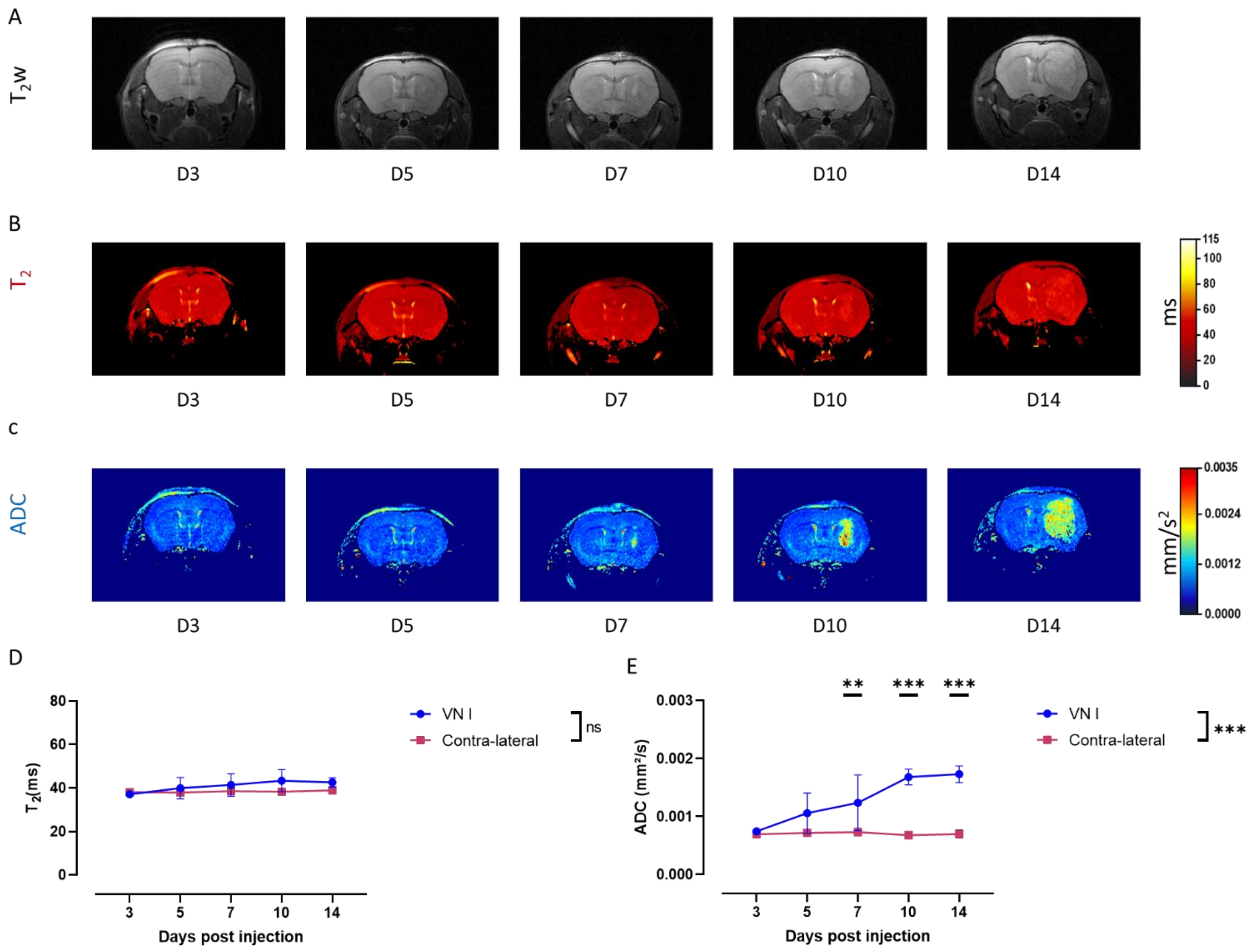


**Figure S3. VN I longitudinal qMRI assessment of lesion (right striatum) and contralateral side (left striatum).** $T_2$w (A), $T_2$ maps (B) and ADC maps (C) from one animal acquired at the different time points. The graphs show the average and standard deviation of $T_2$ Values (D) and ADC values (E) of the lesion and corresponding contralateral side. 2-way ANOVA with Tukey's multiple comparison. (p-values: ns>0.05, * < 0.05, ** < 0.01, *** < 0.001, **** < 0.0001).

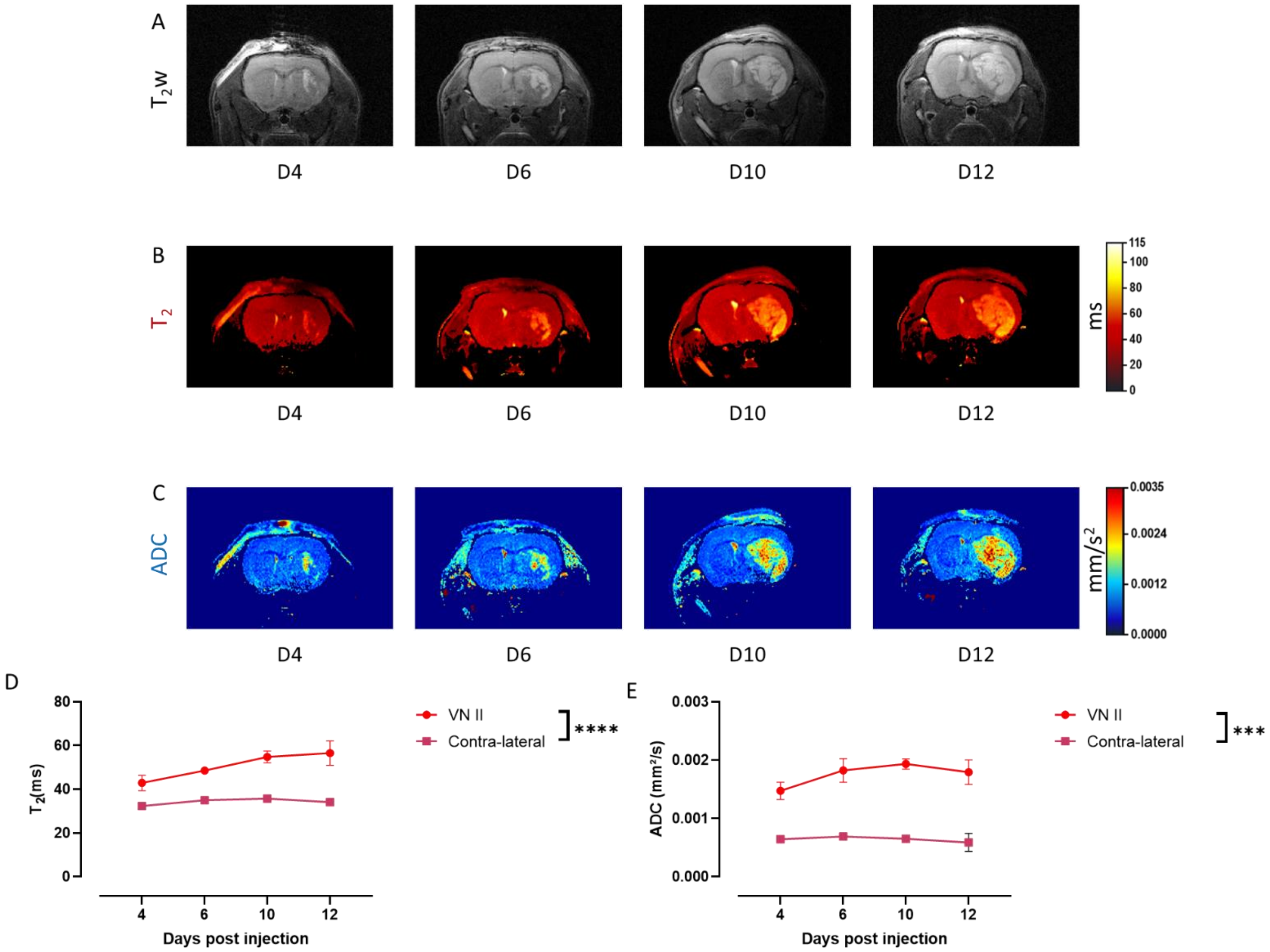


**Figure S4. VN II longitudinal qMRI assessment of lesion (right striatum) and contralateral side (left striatum).** $T_2$w (A), $T_2$ maps (B) and ADC maps (C) from one animal acquired at the different time points. The graphs show the average and standard deviation of $T_2$ Values (D) and ADC values (E) of the lesion and corresponding contralateral side. 2-way ANOVA with Tukey's multiple comparison. (p-values: ns>0.05, * < 0.05, ** < 0.01, *** < 0.001, **** < 0.0001).

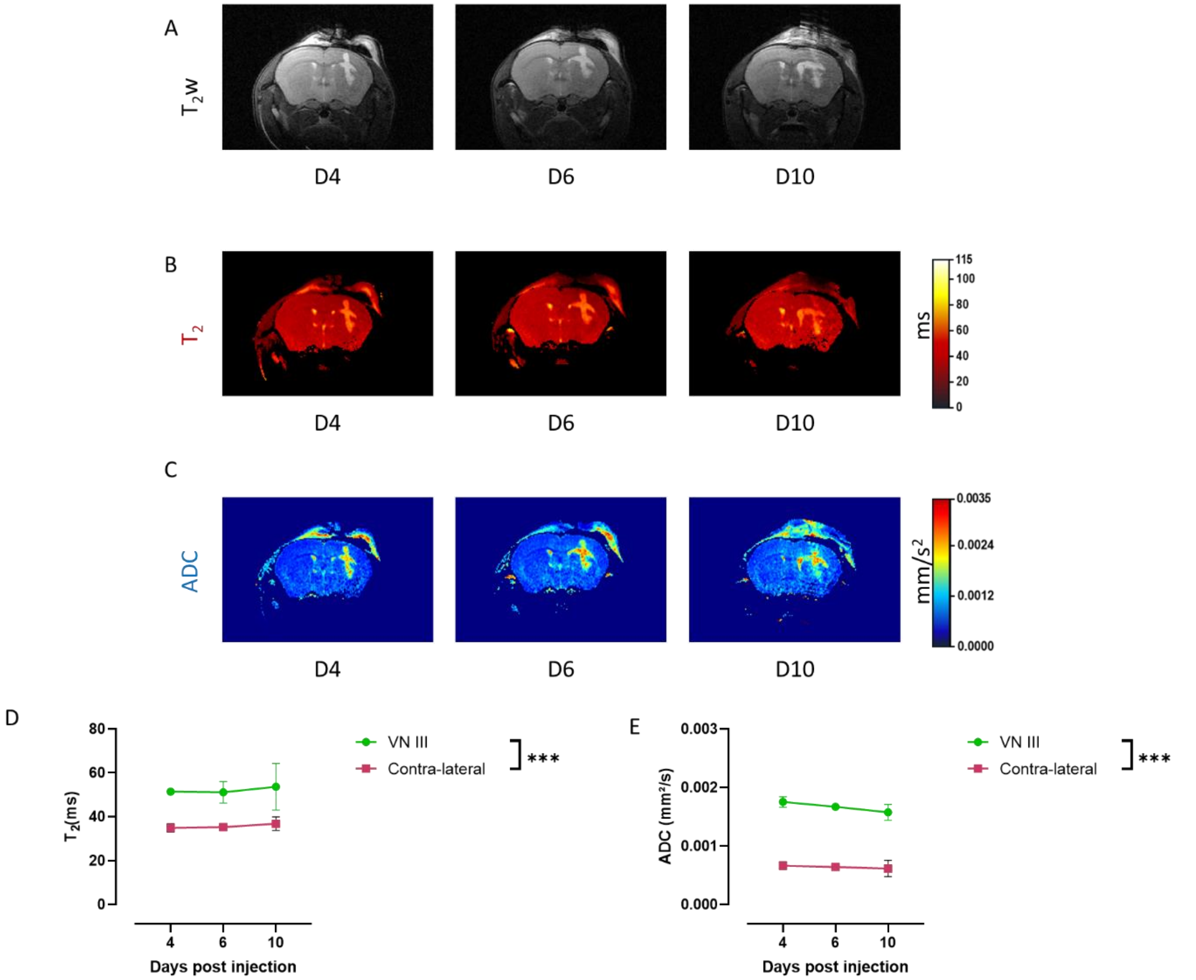


**Figure S5. VN III longitudinal qMRI assessment of lesion (right striatum) and contralateral side (left striatum).** $T_2$w (A), $T_2$ maps (B) and ADC maps (C) from one animal acquired at the different time points. The graphs show the average and standard deviation of $T_2$ Values (D) and ADC values (E) of the lesion and corresponding contralateral side. 2-way ANOVA with Tukey's multiple comparison. (p-values: ns>0.05, * < 0.05, ** < 0.01, *** < 0.001, **** < 0.0001).

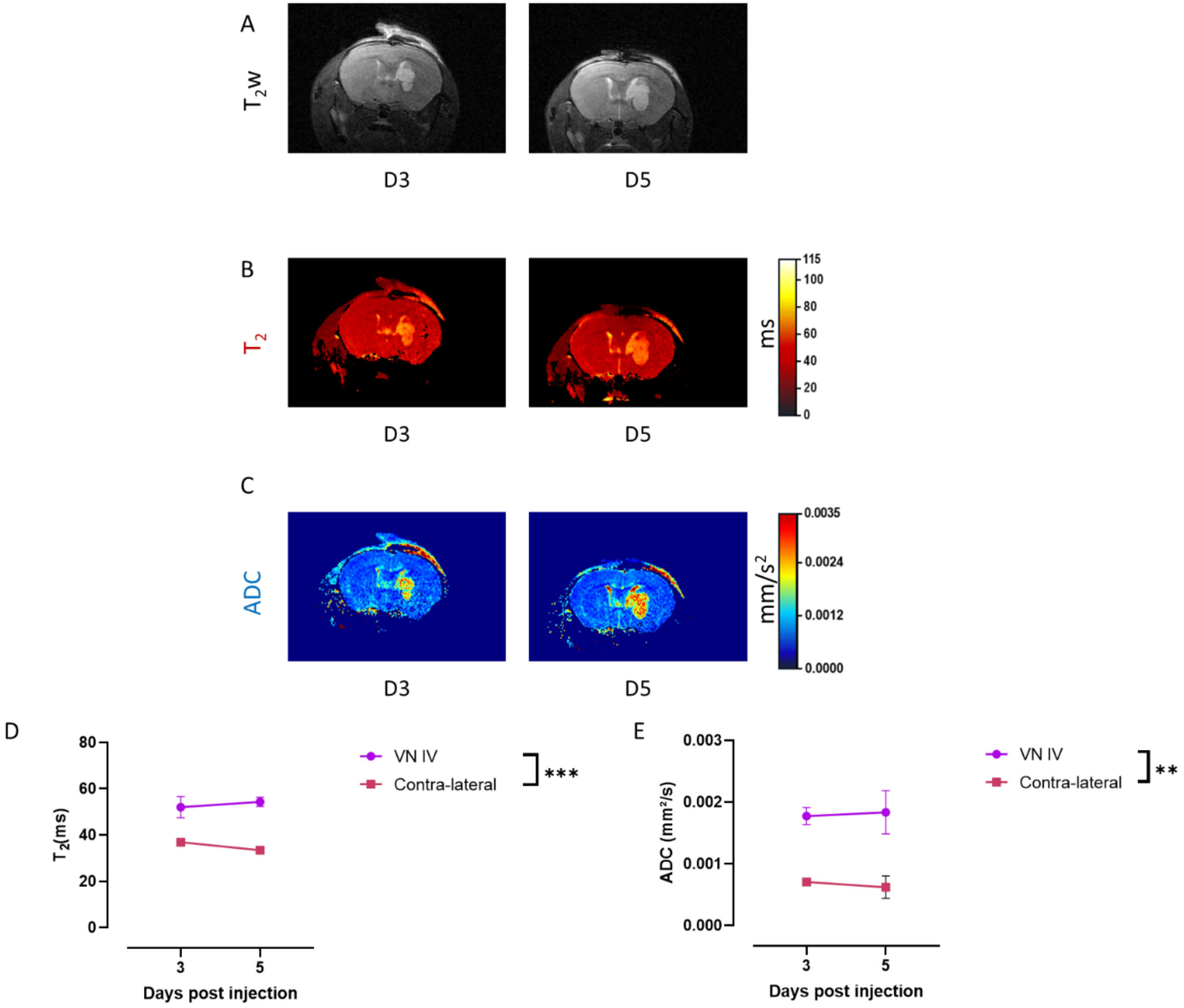


**Figure S6. VN IV longitudinal qMRI assessment of lesion (right striatum) and contralateral side (left striatum).** $T_2$w (A), $T_2$ maps (B) and ADC maps (C) from one animal acquired at the different time points. The graphs show the average and standard deviation of $T_2$ Values (D) and ADC values (E) of the lesion and corresponding contralateral side. 2-way ANOVA with Tukey's multiple comparison. (p-values: ns>0.05, * < 0.05, ** < 0.01, *** < 0.001, **** < 0.0001).

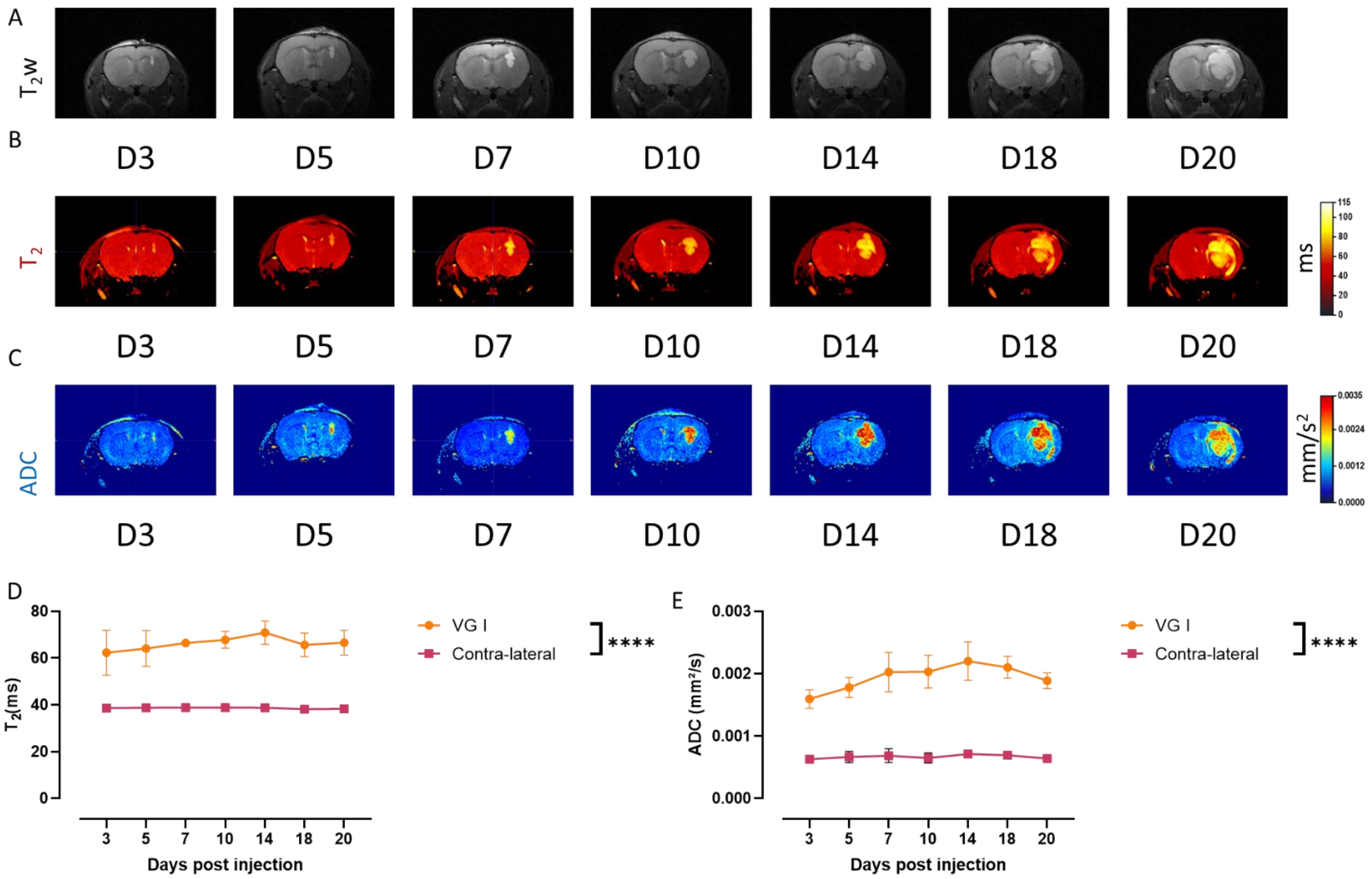


**Figure S7. VG I longitudinal qMRI assessment of lesion (right striatum) and contralateral side (left striatum).** $T_2w$ (A), $T_2$ maps (B) and ADC maps (C) from one animal acquired at the different time points. The graphs show the average and standard deviation of $T_2$ Values (D) and ADC values (E) of the lesion and corresponding contralateral side. 2-way ANOVA with Tukey's multiple comparison. (p-values: ns>0.05, * < 0.05, ** < 0.01, *** < 0.001, **** < 0.0001).

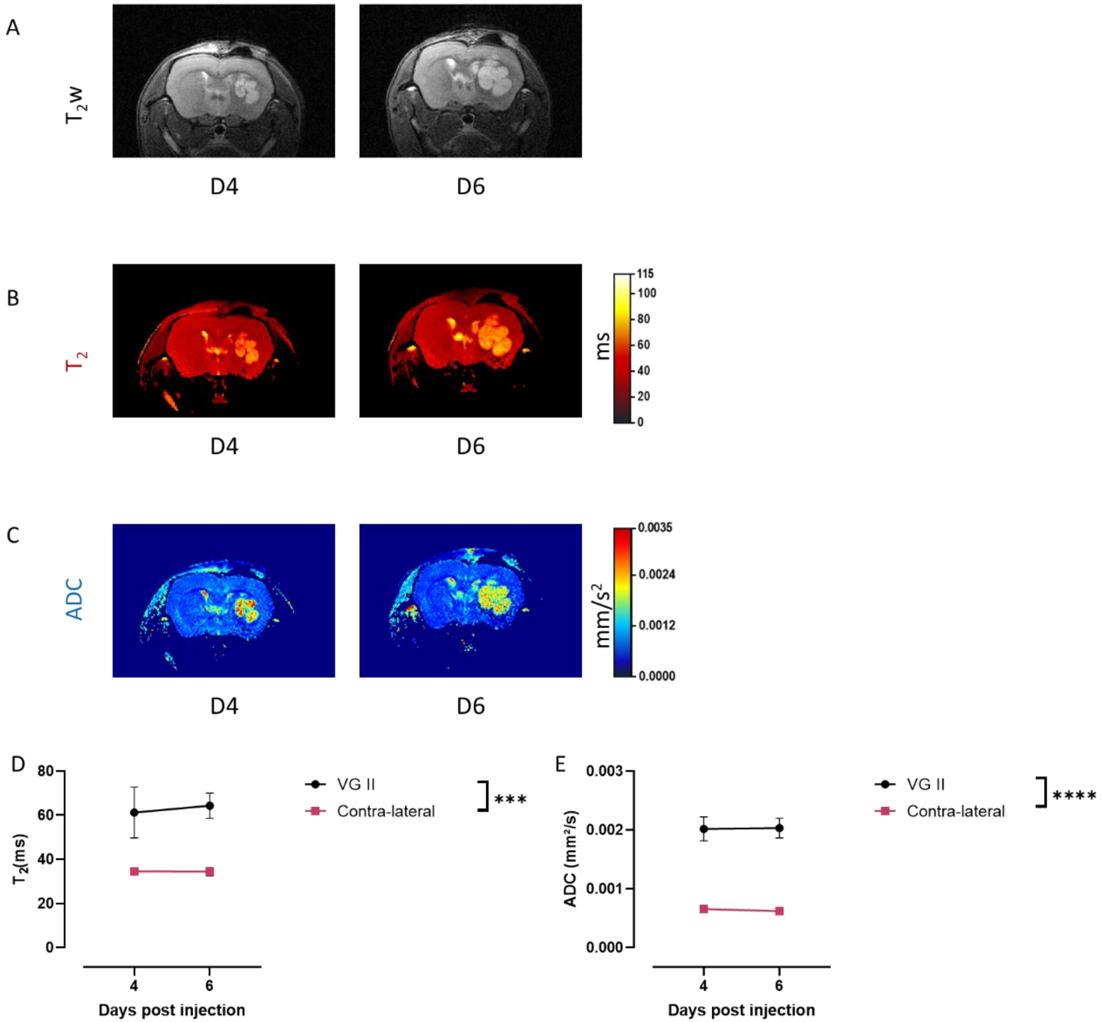


**Figure S8. VG II longitudinal qMRI assessment of lesion (right striatum) and contralateral side (left striatum).** $T_2$w (A), $T_2$ maps (B) and ADC maps (C) from one animal acquired at the different time points. The graphs show the average and standard deviation of $T_2$ Values (D) and ADC values (E) of the lesion and corresponding contralateral side. 2-way ANOVA with Tukey's multiple comparison. (p-values: ns>0.05, * < 0.05, ** < 0.01, *** < 0.001, **** < 0.0001).

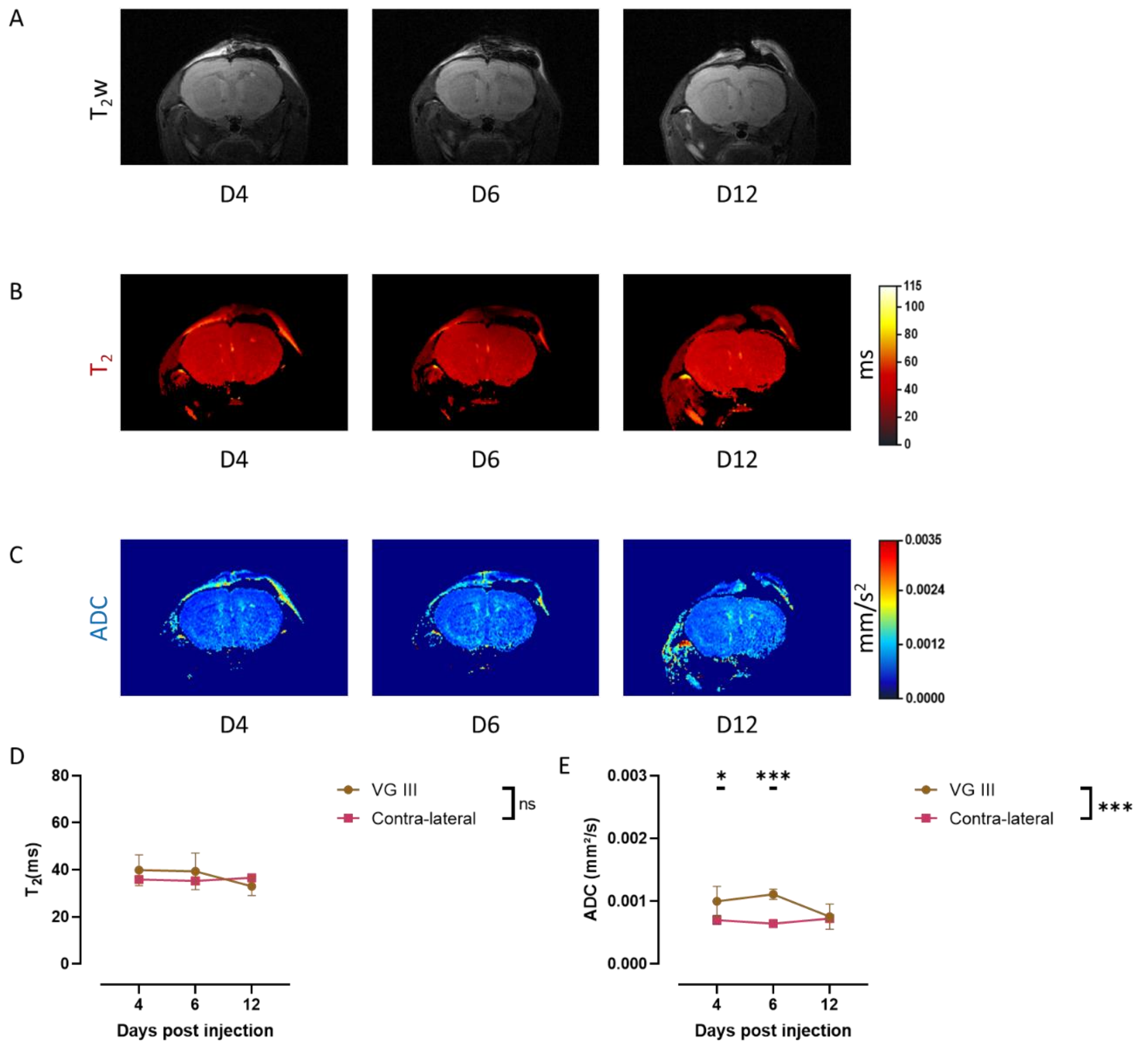


**Figure S9. VG III longitudinal qMRI assessment of lesion (right striatum) and contralateral side (left striatum).** $T_2$w (A), $T_2$ maps (B) and ADC maps (C) from one animal acquired at the different time points. The graphs show the average and standard deviation of $T_2$ Values (D) and ADC values (E) of the lesion and corresponding contralateral side. 2-way ANOVA with Tukey's multiple comparison. (p-values: ns>0.05, * < 0.05, ** < 0.01, *** < 0.001, **** < 0.0001).

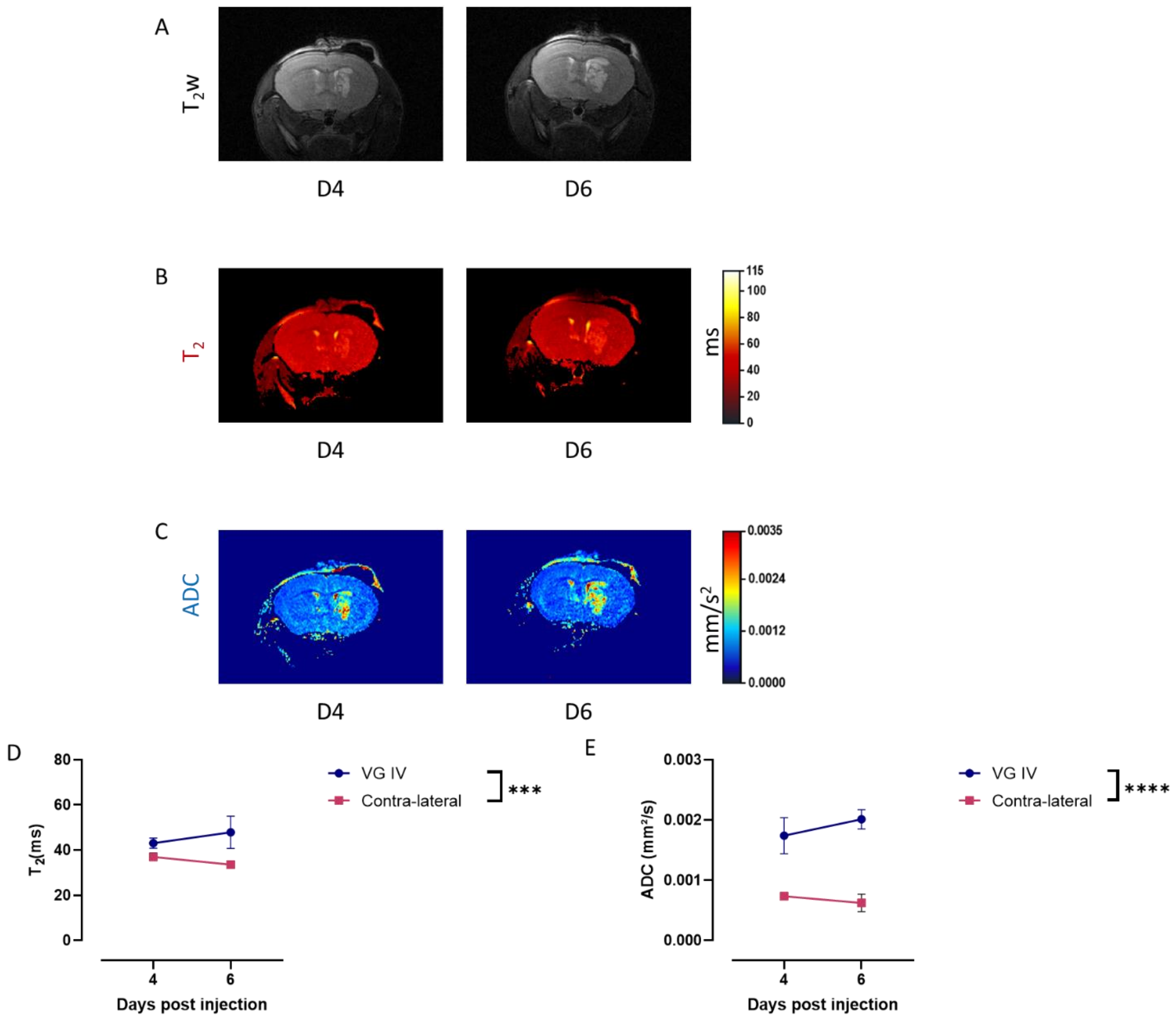


**Figure S10. VG IV longitudinal qMRI assessment of lesion (right striatum) and contralateral side (left striatum).** $T_2$w (A), $T_2$ maps (B) and ADC maps (C) from one animal acquired at the different time points. The graphs show the average and standard deviation of $T_2$ Values (D) and ADC values (E) of the lesion and corresponding contralateral side. 2-way ANOVA with Tukey's multiple comparison. (p-values: ns>0.05, * < 0.05, ** < 0.01, *** < 0.001, **** < 0.0001).

**References**

1. Meyer, W.; Aanensen, D.M.; Boekhout, T.; Cogliati, M.; Diaz, M.R.; Esposto, M.C.; Fisher, M.; Gilgado, F.; Hagen, F.; Kaocharoen, S.; et al. Consensus Multi-Locus Sequence Typing Scheme for Cryptococcus Neoformans and Cryptococcus Gattii. *Med Mycol* **2009**, *47*, 561–570, doi:10.1080/13693780902953886.
2. Engelthaler, D.M.; Hicks, N.D.; Gillece, J.D.; Roe, C.C.; Schupp, J.M.; Driebe, E.M.; Gilgado, F.; Carriconde, F.; Trilles, L.; Firacative, C.; Ngamskul-rungroj, P.; Castañeda, E.; dos Santos Lazera, M.; Melhem, M.S.C.; Pérez-Bercoff Å; Huttley, G.; Sorrell, T.C.; Voelz, K.; May, R.C.; Fisher, M.C.; Thompson III, G.R.; Lockhart, S.R.; Keim, P.; Meyer, W. *Cryptococcus gattii* in North American Pacific Northwest: Whole-Population Genome Analysis Provides Insights into Species Evolution and Dispersal. *mBio* **2014**, *5*, e01464-14, doi:10.1128/mBio.01464-14.
3. Fernandes, K.E.; Dwyer, C.; Campbell, L.T.; Carter, D.A. Species in the *Cryptococcus gattii* Complex Differ in Capsule and Cell Size following Growth under Capsule-Inducing Conditions. *mSphere* **2016**, *1*, e00350-16, doi:10.1128/mSphere.00350-16.
